# Translation of EEG Spatial Filters from Resting to Motor Imagery Using Independent Component Analysis

**DOI:** 10.1371/journal.pone.0037665

**Published:** 2012-05-29

**Authors:** Yijun Wang, Yu-Te Wang, Tzyy-Ping Jung

**Affiliations:** 1 Swartz Center for Computational Neuroscience, Institute for Neural Computation, University of California San Diego, San Diego, California, United States of America; 2 Institute of Engineering in Medicine, University of California San Diego San Diego, California, United States of America; Cuban Neuroscience Center, Cuba

## Abstract

Electroencephalogram (EEG)-based brain-computer interfaces (BCIs) often use spatial filters to improve signal-to-noise ratio of task-related EEG activities. To obtain robust spatial filters, large amounts of labeled data, which are often expensive and labor-intensive to obtain, need to be collected in a training procedure before online BCI control. Several studies have recently developed zero-training methods using a session-to-session scenario in order to alleviate this problem. To our knowledge, a state-to-state translation, which applies spatial filters derived from one state to another, has never been reported. This study proposes a state-to-state, zero-training method to construct spatial filters for extracting EEG changes induced by motor imagery. Independent component analysis (ICA) was separately applied to the multi-channel EEG in the resting and the motor imagery states to obtain motor-related spatial filters. The resultant spatial filters were then applied to single-trial EEG to differentiate left- and right-hand imagery movements. On a motor imagery dataset collected from nine subjects, comparable classification accuracies were obtained by using ICA-based spatial filters derived from the two states (motor imagery: 87.0%, resting: 85.9%), which were both significantly higher than the accuracy achieved by using monopolar scalp EEG data (80.4%). The proposed method considerably increases the practicality of BCI systems in real-world environments because it is less sensitive to electrode misalignment across different sessions or days and does not require annotated pilot data to derive spatial filters.

## Introduction

In electroencephalogram (EEG)-based brain-computer interface (BCI) research, the motor imagery-based BCI has attracted much attention in the past two decades [1]. A motor imagery-based BCI translates a subject's motor intention into a command signal through detecting motor imagery states (e.g., imagination of left and right hand movements) in near-real time. Pfurtscheller *et al.* [2], [3] developed the first motor imagery-based BCI based upon the detection of EEG power changes, known as Event-Related Desynchronization and Synchronization (ERD/ERS), in movement-related mu (8–12 Hz) and beta (18–26 Hz) rhythms. Wolpaw *et al.* [4] proposed another motor imagery-based approach to train the users to regulate the amplitude of mu/beta rhythms to control a 2-D cursor movement. Compared to other commonly used EEG signals such as the event-related P300 potential and visual evoked potentials (VEPs), the motor imagery-based BCI does not require external stimuli and could be totally independent of muscle activities [4]; therefore, it is more acceptable to the users.

Currently, machine-learning techniques play an important role in implementing a motor imagery-based BCI [5], [6]. Because EEG changes during motor imagery are subject-specific in both frequency and spatial domains, a calibration is required for collecting labeled data to optimize spatial filters and classifiers for each individual [7]. Furthermore, other recording parameters (e.g., electrode position, skin contact, and system noise) and the non-stationarity of EEG signals also pose a challenge to re-calibrate the system across different sessions even for the same user. The calibration procedure is always labor and time consuming, and therefore, seriously limits the practicality of BCIs in real-world environments.

Recently, several motor imagery-based BCI studies have developed adaptive methods or zero-training methods to resolve the problems caused by session-to-session and subject-to-subject variability [8]–[10]. These methods were proposed based on assumptions that there are common EEG spatial patterns across sessions within subjects and across different subjects. Considering large variability in anatomy across subjects, the spatial filters derived from a subject might not be optimal for another. Furthermore, searching reproducible session-to-session spatial filters remains to be labor and time expensive because a good labeled pilot data for each individual is required. An alternative solution is to find stable state-to-state spatial filters, which could be obtained by a short pilot session without requiring labor-intensive labeling, such as a few minutes of resting EEG. The underlying assumption in the state-to-state solution is that the spatial patterns of some function-specific EEG components (e.g., motor components) are relatively stable from one state to another. In a motor imagery-based BCI, it is reasonable to assume that spatial patterns of the mu/beta components are consistently located in the primary sensorimotor cortex under both the resting state and the motor imagery state for each subject. Based on this hypothesis, it might be possible to derive spatial filters based on non-labeled EEG data recorded during a resting state and apply them to the classification of EEG data during motor imagery BCI practices.

To realize a rest-to-work translation of EEG spatial filters, two preconditions have to be met. First, EEG sources are spatially stable in both the resting and working states and can be reflected by detectable EEG oscillations. Second, the method used for learning spatial filters is fully unsupervised so that the training data do not require any labor-intensive annotation. Previous studies have shown that EEG changes during motor imagery of hand movements are pre-dominated by EEG power modulations of the mu/beta rhythms (ERD/ERS) in the hand area, which is the largest subarea in the sensorimotor cortex [2]. Previous studies have also reported brain activities from default brain networks when the brain stays in its idling state [11], [12]. To be more specific, the spontaneous mu/beta rhythms often show strong activities during resting, indicating the idling of the primary sensorimotor cortex [13]. Therefore, the hypothesis of this study is that spatial patterns of EEG changes of the mu/beta rhythms in the resting state are consistent with those associated with imagery of hand movements. The advantages of using spatial filters from resting EEG are two-fold: (1) it does not require labor-intensive labeling; (2) the pilot data are readily available without requiring subjects' attentions or actions.

An unsupervised method is needed to find spatial filters from unlabeled resting EEG data. Independent component analysis (ICA) is a practical solution because it can decompose overlapped brain source activities constituting the scalp EEG into functionally specific components [14]. Furthermore, many studies applying ICA to decompose sets of averaged ERPs, continuous EEG records, and/or sets of event-related EEG data trials have demonstrated that independent motor components were very consistent in terms of their scalp projections and spectral profiles across different mental tasks [15]–[17]. We thus hypothesize that ICA, applied to unlabeled resting EEG, may find spatial filters for discriminating different motor imagery states.

The goal of this study is to investigate the feasibility of deriving the state-invariant EEG spatial filters, based on resting EEG, for a motor imagery-based BCI. The accuracies of using spatial filters derived from resting EEG to classify single-trial EEG during imagining left and right hand movements are compared to those using the spatial filters derived from data collected in the motor imagery state. In addition, this study also compares the proposed state-to-state method to the existing session-to-session method [10].

## Methods

### 1 Experimental paradigm and data recording

**Figure 1:**
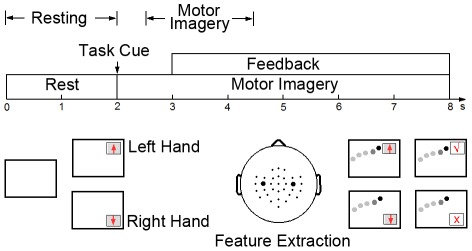
Experiment paradigm for the motor imagery-based brain-computer interface.

The dataset used in this study was provided by the Institute of Neural Engineering at Tsinghua University [18]. Nine healthy volunteers participated in an online BCI experiment. Figure 1 shows the paradigm of motor imagery-based BCI control with visual feedback. The left- and right-hand movement imaginations were designated to control vertical cursor movement on the screen. The subject sat comfortably in an armchair, facing a computer screen displaying visual feedback. The duration of each trial was 8 seconds. During the first 2 seconds, while the screen was blank, the subject was in the resting state. Immediately after these first 2 seconds, a visual cue (arrow) was presented on the screen, indicating the imagery task to be performed. The arrows pointing upwards and downwards indicated the imagination of the left hand and the right hand movement, respectively. After 3 seconds, a cursor started to move at a constant speed from the left side to the right side of the screen. The vertical position of the cursor was determined by the power difference of mu rhythm between the left and right hemispheres (C3 and C4 electrodes). After 8 seconds, a true or false mark appeared on the screen to indicate the final result of the trial and the subject was asked to relax and wait for the next task. At the beginning of a block, an adaptive method was employed to optimize the classifier with the first 10 trials (5 trials per class) [18].

32-channel EEG signals referenced to the CMS-DRL ground (see www.biosemi.com/faq/cms&drl.htm for more information) were recorded using a BioSemi ActiveTwo system with scalp electrodes placed according to the modified 10–20 international system. The signals were digitized at 256 Hz and band-pass filtered (2–30 Hz) for further analysis. For each subject, the experiment consisted of four blocks each including 60 trials (30 trials per class). There were 3–5 minutes breaks between two consecutive blocks. A total of 240 trials (120 trials per class) were recorded in one session for each subject. Three of the nine subjects (S5, S6, and S8) participated in a second session on a different day using the same experimental setup. The intervals between two sessions were more than 3 weeks (26, 75, and 35 days for the three subjects respectively).

### 2 Data processing and analysis

#### 2.1 Independent component analysis

ICA is a statistical method that aims to find linear projections of the observed data that maximize their independence [19]. When applied in Blind Source Separation (BSS), ICA aims to recover independent sources using multi-channel observations of mixtures of those sources. In the past two decades, ICA has been successfully used in processing biomedical signals including EEG, electrocardiogram (ECG), magnetoencephalogram (MEG), and functional magnetic resonance imaging (fMRI) signals [20]. In EEG signal processing, ICA has shown a good capability in separating the scalp EEG signals into functionally independent sources, including neural components originating from different brain areas and artifactual components attributed to eye movements, blinks, muscle, heart, and line noise. Due to its superiority in EEG source separation, ICA has been successfully applied to many EEG research fields including artifact removal, signal-to-noise ratio (SNR) enhancement of task-related EEG signals, and EEG source localization [21]. Many EEG-based BCI studies have employed ICA to enhance task-related EEG signals [22], [23], and optimize electrode positions [24], [25].

Given a linear mixing model, *n*-channel Scalp EEG signals 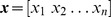 are generated by *m* independent sources 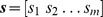:
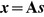
(1)where **A** is the 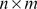 mixing matrix in the model. After ICA, source signals can be estimated by applying an unmixing matrix 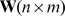 to the observed EEG data 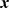:
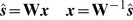
(2)where each row of **W** is a spatial filter for estimating an independent component and each column of **W^−1^** consists of electrode weights (i.e., spatial projection) of an independent component.

As indicated in Figure 1, the 0–2 s and 2.5–4.5 s segments in a trial were selected to represent the resting state and the motor imagery state, respectively. For each subject, ICA was performed on data under the two states separately. For each state, data of all trials were concatenated to a 480-second (240 trials×2 seconds) long data segment. ICA was performed using the EEGLAB toolbox with the extended infomax algorithm [26]. 32-channel data were first projected to a 15-dimensional subspace using principal component analysis (PCA). This study used PCA to reduce the dimensionality of the data due to the following two reasons: (1) PCA can improve the robustness of ICA, which was unable to achieve stable decompositions in different runs because of the small size of data in this study (480 seconds). (2) PCA can significantly reduce the computation time and the need of large amounts of computer memory, which in turn improves the practicality of the proposed method in online applications. In these data sets, the number of typical EEG components ranged from 8 to 15 (mean: 11.2±2.9). To guarantee the extraction of all brain components for all subjects, this study used 15 PCs as inputs to the ICA. Then, for each subject, ICA resulted in two sets of 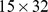 spatial filters (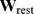 and 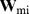) and 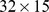 spatial projections (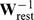 and 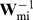) corresponding to the resting and motor imagery states.

#### 2.2 Identifying motor components

In previous studies, ICA has shown its robustness in finding motor components, which have characteristic features in spatial and frequency domains [14], [15], [26], [27]. This study used two criteria to identify motor-related components after ICA: (1) the spatial pattern, which suggests the source location of the component, should be consistent with the scalp projection of the sensorimotor cortex on each hemisphere; (2) the power spectrum density (PSD) of the component should match the typical spectral profile of mu/beta rhythms. In practice, a motor component should fit both criteria. Previous studies showed that the number of motor components varied across subjects [14], [17]. On one hand, there might be multiple motor-like ICs on one hemisphere; on the other hand, motor components might be missing on some people. In this study, all subjects were able to control the BCI by regulating the amplitude of the mu rhythm on both hemispheres. Therefore, two typical motor ICs, which represented brain activities originating from the left and right motor areas, were expected to be extracted by ICA. In this study, ICA extracted two motor ICs for eight subjects and three motor ICs (two left ICs and one right IC) for one subject (Subject 5). This study focused on the state-to-state translation approach for obtaining EEG spatial filters. Hence, without loss of generality, this study only selected two ICs, one for each hemisphere, for further data analysis. In addition, Subsection 4 in the discussion section will present the result of classification involving all three motor ICs for Subject 5.

For the purpose of online implementation, this study developed an automatic approach for identifying the motor components. The motor ICs were first selected manually according to the aforementioned criteria, providing an objective basis for evaluating the proposed method. This study defined three quantitative parameters to characterize the motor IC on each hemisphere: (1) distance between the equivalent dipole, which was obtained using DIPFIT plugin in EEGLAB [26], and the group mean of dipoles of the motor component; (2) correlation between IC's spatial pattern and the mean of spatial patterns of the motor ICs across all subjects; (3) EEG power ratio of the mu rhythm (10–15 Hz) to its neighbors in the frequency band of 15–20 Hz. For each parameter, an index could be obtained for each IC by sorting the values of the parameter across all ICs. The index reflects the similarity between an IC and a motor component (a smaller index value indicates a higher similarity to a motor component, i.e., smaller distance, higher correlation, and larger power ratio). The identification of the motor components combined these parameters together to calculate a motor index (*f*) as follows:
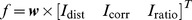
(3)where *I*_dist_, *I*_corr_, and *I*_ratio_ are the indices corresponding to the three parameters. The weights for the three parameters (***w***) were determined toward identifying the same ICs as those selected manually. This study used [3 1 2] as the weighting vector to adjust the contributions of the three parameters. The left and the right motor IC was considered separately. The IC, which had the smallest value of *f*, was selected as the motor IC. ICs with residual variance (RV) for the dipole fit higher than 20% were rejected before this process.

**Figure 2:**
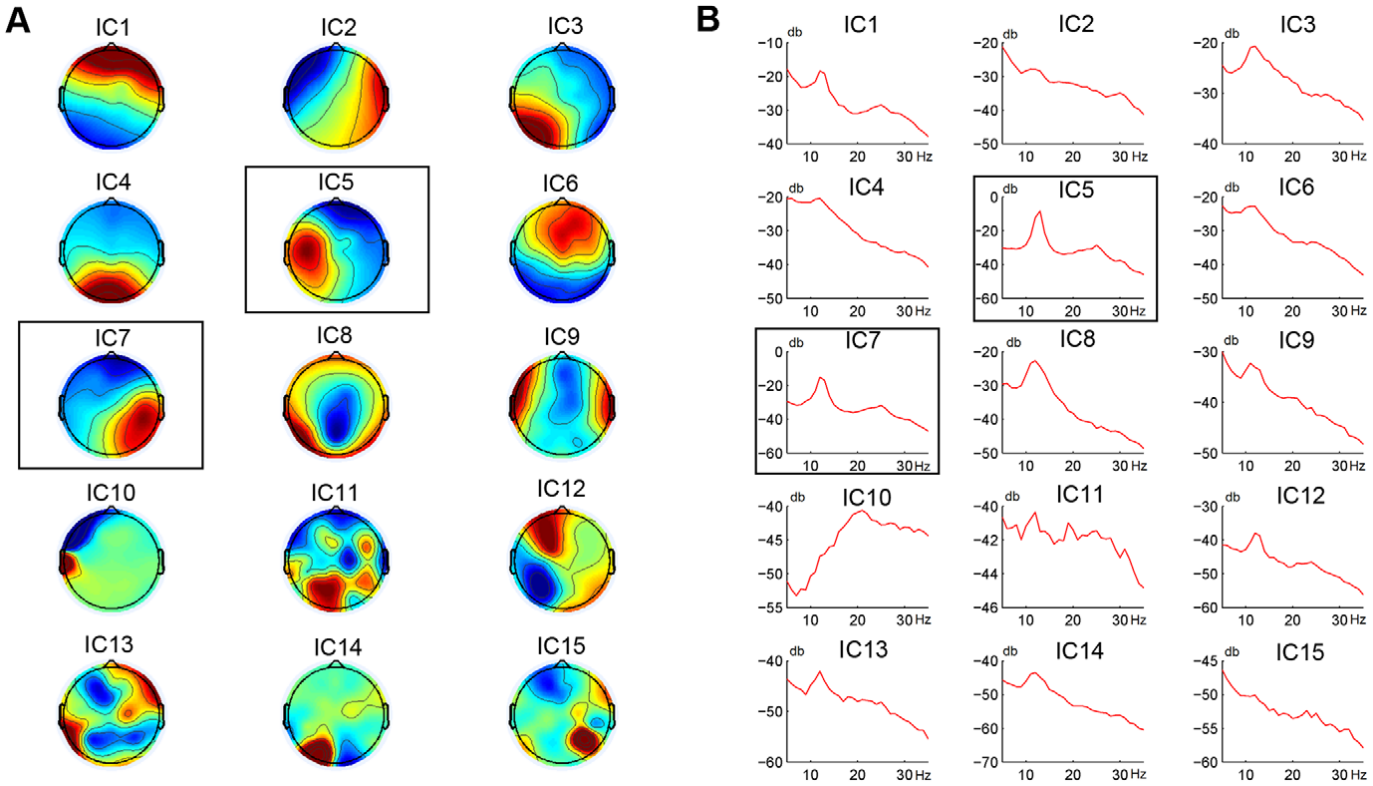
Scalp topographies and PSDs of all ICs from one subject. (A) Scalp topographies; (B) PSDs. IC5 and IC7, which both show a unilateral spatial distribution over the sensorimotor cortex and a mu/beta-band dominant spectral profile, are highlighted by a black rectangle as the selected motor components (cf. details of the identification process in Table 1).

This study used a leave-one-out method to calculate the first two parameters for each subject (i.e., the group means of the dipole location and the spatial pattern of the motor component were obtained without the subject's own data). For all subjects, the ICs identified by this quantitative approach were exactly the same as those selected manually. Figure 2 shows spatial projections and PSDs of all independent components based on the EEG data collected in the motor imagery state for a sample subject. Table 1 lists all parameters used for identifying the motor components. ICs 11, 12, 13, and 15 were rejected first because of their high RVs in dipole fitting (>20%). According to the calculated motor index, IC5 and IC7, which had the smallest index value (i.e., *f*  =  6 and 8 for the left and the right motor IC, respectively), were identified as the two motor components. As shown in Figure 2, they both have a unilateral spatial distribution over the sensorimotor cortex, as well as a mu/beta-band dominant spectral profile.

**Table 1:** Parameters for automatic identification of motor ICs on a sample subject.

			Left Motor IC			Right Motor IC		
IC Index	RV (%)	Power Ratio (*I*_ratio_)	Distance (*I*_dist_)	Correlation (*I*_corr_)	*f*(*I*)	Distance (*I*_dist_)	Correlation (*I*_corr_)	*f*(*I*)
1	2.2	2.3 (4)	88 (8)	0.54 (4)	36 (5)	92 (5)	0.07 (9)	32 (4)
2	0.7	1.3 (10)	100 (10)	0.58 (3)	53 (10)	115 (9)	0.81 (2)	49 (9)
3	1.3	1.7 (8)	90 (9)	0.76 (2)	45 (8)	111 (8)	0.44 (4)	44 (8)
4	1.0	1.8 (7)	82 (7)	0.18 (10)	45 (8)	84 (4)	0.14 (7)	33 (5)
5	3.1	6.3 (1)	21 (1)	0.95 (1)	6 (1)	67 (3)	0.53 (3)	14 (2)
6	2.8	2.1 (5)	48 (2)	0.42 (6)	22 (3)	39 (2)	0.30 (5)	21 (3)
7	2.5	4.8 (2)	71 (4)	0.43 (5)	21 (2)	11 (1)	0.94 (1)	8 (1)
8	14.2	2.8 (3)	78 (5)	0.27 (7)	28 (4)	109 (7)	0.08 (8)	35 (6)
9	6.4	1.6 (9)	131 (11)	0.23 (8)	59 (11)	123 (11)	0.27 (6)	57 (10)
10	7.8	0.5 (11)	60 (3)	0.22 (9)	40 (6)	117 (10)	0.03 (11)	63 (11)
11	54.6	-	-	-	-	-	-	-
12	32.3	-	-	-	-	-	-	-
13	53.4	-	-	-	-	-	-	-
14	10.2	1.9 (6)	80 (6)	0.05 (11)	41 (7)	103 (6)	0.07 (10)	40 (7)
15	78.7	-	-	-	-	-	-	-

#### 2.3 ERD/ERS during motor imagery

**Figure 3:**
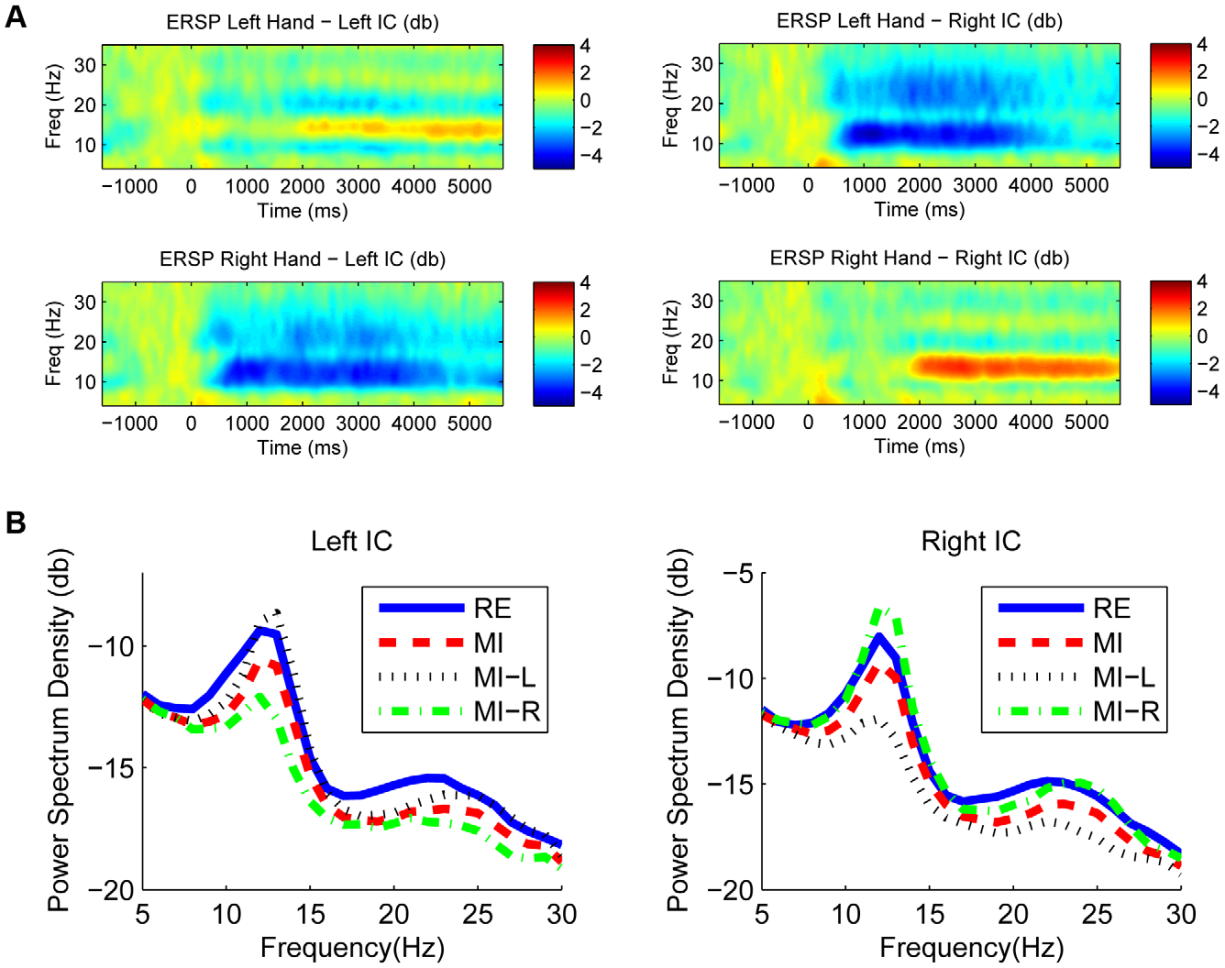
Group-averaged ERSP and PSD for two motor components. (A) Group-averaged time-frequency distributions of ERSP for the left motor IC and the right motor IC corresponding to left and right hand movement imaginations; (B) Group-averaged PSD of left and right motor ICs under different conditions (RE: resting state, MI: motor imagery state, MI-L: left-hand motor imagery, MI-R: right-hand motor imagery).

As mentioned before, if the mu/beta rhythms are detectable in both the resting state and the motor imagery state, then, it is feasible to translate ICA-based spatial filters from the resting state to the motor imagery state. Two well-known phenomena about ERD/ERS of the mu/beta rhythms have been reported in previous motor imagery studies: a contralateral ERD and an ipsilateral ERS, both with respect to the imagined hand movements. The contralateral ERD indicates the excitation of the hand area corresponding to the imagined hand, whereas the ipsilateral ERS shows the inhibition of the hand area corresponding to the resting hand [28]. Figure 3A shows the group-averaged event-related spectral perturbation (ERSP) of the motor components in the motor imagery state using the resting state as a baseline. During motor imagery, a significant mu/beta ERD occurred over the hemisphere contralateral to the imagined hand. An ERS on the ipsilateral hemisphere also existed at a late stage of the motor imagery period. Figure 3B shows PSDs of the motor components extracted by applying ICA-based spatial filters derived from the motor imagery state. Both the resting and motor imagery states show a typical spectral profile peaking at the mu/beta frequency band, although the motor imagery state has an overall power decrease. When considering motor imagery of the left hand and the right hand separately in each component, the ERD induced by the contralateral hand movement is stronger than the ERS induced by the ipsilateral hand. The mu/beta rhythms appear dominant in the PSDs under both the resting state and the motor imagery state (Figure 3B); ICA thus could extract independent motor-related activities from the scalp EEG data.

### 3 Translating spatial filters from resting to BCI practice

As mentioned above, to realize a state-to-state translation of EEG spatial filters in a motor imagery BCI, EEG sources of motor activities need to be spatially stable in both states and can be reflected by detectable EEG oscillations. Because the two motor components in the resting state and the motor imagery state have strong similarities in EEG PSDs (Figure 3B) and spatial patterns, it might be feasible to use the spatial filters obtained in the resting state as estimates of the spatial filters in the motor imagery state.

**Figure 4:**
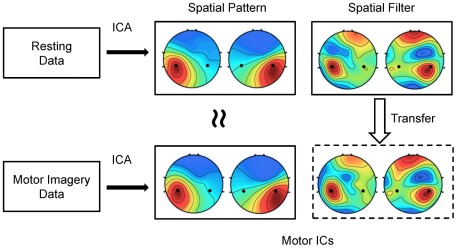
Diagram of translating spatial filters from the resting state to the motor imagery state. Similar spatial filters and spatial patterns were obtained by ICA on data corresponding to the two conditions separately. Spatial filters obtained from the resting data could be used as estimates of those from the motor imagery data.

The proposed method aimed to translate EEG spatial filters from the resting state to the motor imagery state. The basic procedure can be described as follows:
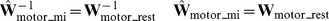
(4)where 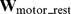 and 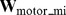 are motor-related spatial filters for the resting state and the motor imagery state respectively. 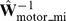 and 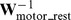 are the corresponding spatial patterns to the two conditions. Figure 4 illustrates the principle of the proposed method. When considering separately, ICA can obtain spatial filters from data under both conditions. In Figure 4, the scalp distributions of spatial patterns and spatial filters of selected motor ICs for a sample subject show very high similarities. Because data in the resting state and the motor imagery state are totally non-overlapped, the spatial filters derived from the resting data could be used as estimates of the spatial filters for the motor imagery data. In practice, the resting EEG data, which do not require the subject's attention or action, can be easily collected before a BCI session, and therefore, can facilitate the user training procedure required for optimizing EEG spatial filters.

### 4 Feature extraction and classification

This study compares the classification performance of the motor-imagery BCI based on three different types of EEG features listed below.

#### 4.1 Monopolar scalp EEG data

Motor imagery of the left and the right hand movement results in different spatial distributions of mu EEG power over the sensorimotor brain areas. To make a direct comparison with the ICA-based spatial filtering method, electrodes C3 and C4, which represented the left and the right sensorimotor areas on both hemispheres, were selected for feature extraction. This study used band-pass EEG power as features for classification. To measure EEG power, Fast Fourier Transform (FFT) with a rectangular window function converted the data segments during the motor imagery state (2.5–4.5 s in each trial) into frequency-domain responses for each channel. The resultant features for classification were accumulated EEG power between 8 and 30 Hz at C3 and C4:
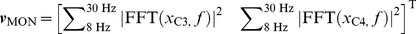
(5)


#### 4.2 ICA-based spatial filtering

After ICA, the selected motor-related unmixing vectors (i.e., 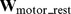 in Equation (4)) were used as spatial filters to extract motor-related EEG activities. In this regime, ICA aimed to enhance SNR of motor-related signals. As mentioned before, this study only selected two motor components to represent brain activities from the left and right sensorimotor areas. After automatic identification of the motor ICs, FFT estimated the PSDs of the time courses of the left and right motor components. ICA-based EEG features were defined as follows:
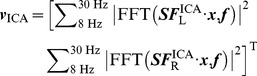
(6)where 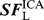 and 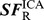 indicated the two spatial filters corresponding to left and right motor components.

This study aimed to evaluate the state-to-state translation of ICA-based spatial filters. ICA was applied separately to the data in the resting state and the motor imagery state; therefore, derived two sets of ICA-based spatial filters (i.e., 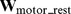 and 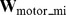). This study further evaluated the performance of the resting-to-work translation method through comparing the classification accuracies of the two ICA-based features corresponding to spatial filters derived from the resting and motor imagery EEG data.

#### 4.3 Common spatial pattern (CSP) based spatial filtering

Previous studies have demonstrated the effectiveness of the CSP algorithm in classifying EEG during motor imagery [29]. To better evaluate the performance of ICA-based filtering, the CSP-based spatial filtering was also conducted in this study. The performance of the CSP method highly depends on subject-specific optimization of time-frequency parameters [30]. Here, for simplicity, the CSP method used the labeled motor imagery data after applying an 8–30 Hz band-pass filter for each subject, resulting in two spatial filters for extracting task-related activities corresponding to the imagination of left and right hand movements. Feature vectors after CSP-based spatial filtering were defined as follows:
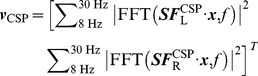
(7)where 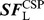 and 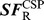 were projection vectors corresponding to the highest and the lowest eigenvalues in the CSP processing [29]. The classification performance often improves as the number of spatial filters increases (e.g., 3 for each class). However, to make a fair comparison between the ICA and the CSP methods, only two CSP filters were used for feature extraction in the present study because only two motor ICs were selected for feature extraction in the ICA-based method.

#### 4.4 Classification

After feature extraction, this study used the Fisher discriminant analysis (FDA) classifier [31] to perform classification. The two-dimensional feature vectors (as shown in Equations (5), (6), and (7)), which represented EEG power over motor areas of both hemispheres, were fed into the FDA classifier. A 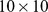-fold cross-validation was used to estimate the classification accuracy for each subject.

For the ICA and the CSP methods, the training of spatial filters used different strategies. The CSP-based spatial filter design used the same cross-validation paradigm (i.e., only training data were used). In the ICA processing, for simplicity, ICA was only run once with the resting data and the motor imagery data separately. After that, the cross-validation procedure used the same ICA-based spatial filters. It is worth mentioning that the resting and the motor imagery data were totally non-overlapped, but the motor imagery data used in ICA training also included data used for testing. Because ICA is an unsupervised learning method, this study assumes that the overlap between ICA training and testing might not overestimate the classification performance of the ICA method using the motor imagery data too much.

### 5 Session-to-session translation

It is important to compare the proposed state-to-state translation method to other zero-training approaches, such as a session-to-session translation method [32]. The session-to-session translation method aims to use information from a pilot session to improve data processing of the subsequent session(s) based on the assumption that there are common EEG patterns across sessions within subjects. Compared to the state-to-state translation, the session-to-session translation may have more challenges due to the long-term non-stationarity of EEG, as well as other parameter changes in data recording (e.g., shift of electrode positions). Moreover, the session-to-session translation is not applicable in the situation where annotated pilot data are not available (e.g., with a naïve subject, or a new system setup). Therefore, a state-to-state translation might be a better solution to optimize zero-training spatial filters for motor-related EEG activities.

To further investigate the feasibility of session-to-session translation, data from the three subjects (S5, S6, and S8), who participated in two separate BCI sessions on different days in this study, were used to evaluate performance of the session-to-session translation method. In both sessions, 32-channel EEG data using the same electrode layouts were recorded with good signal quality. Data process included three procedures: (1) ICA was trained with motor imagery data in the two sessions separately to obtain spatial filters. (2) The ICA-based spatial filters from the first session were translated to the subsequent session from the same subject for processing the data. (3) After applying the session-to-session spatial filters, the classification accuracy of the second session (the same data set used in the state-to-state translation study) was calculated for comparison with other approaches including the monopolar method, the ICA-based method using the same data, and the state-to-state translation method.

## Results

### 1 Similarity between the spatial filters derived from resting and motor- imagery experiments

**Figure 5:**
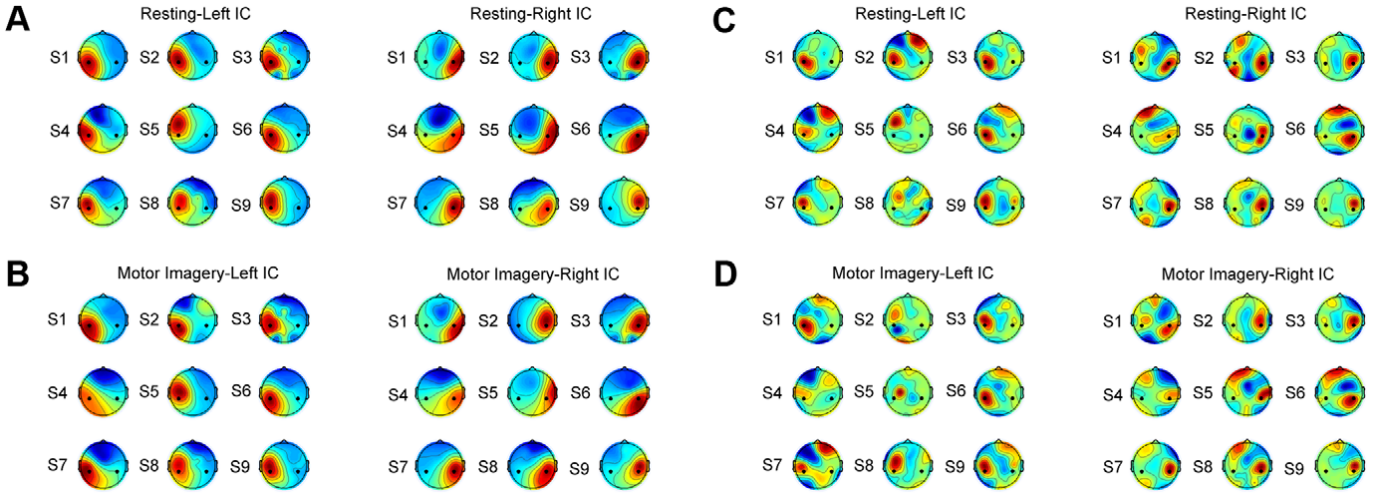
Spatial patterns and spatial filters of the motor components for all nine subjects. (A) spatial patterns of the resting state; (B) spatial patterns of the motor imagery state; (C) spatial filters of the resting state; (D) spatial filters of the motor imagery state. Black dots in each scalp map indicate positions of C3 and C4 electrodes. In each subfigure, the left and right motor ICs for all subjects were grouped on the left and the right panel respectively.

To quantitatively investigate to what extent one can translate the motor-related spatial filters derived from resting to motor-imagery BCI practice, this study first compares spatial patterns and spatial filters of motor components in resting and motor-imagery experiments. Figure 5 shows spatial patterns and spatial filters of the motor components in the resting state and the motor imagery state for all subjects. All the components show a typical dipolar-like topography, which is widespread over the sensorimotor cortex on left or right hemisphere of the brain, and shows the highest amplitudes at C3 and C4 electrodes. These findings are consistent with previous motor-related EEG studies [3]. Generally, the motor-related spatial filters show both positive and negative weights around the sensorimotor area, functioning through eliminating the motor irrelevant background activities while keeping the motor related activities. To quantitatively evaluate the topographical similarity, this study calculated the correlations of spatial patterns and spatial filters of the motor components between the two states for each subject. For simplicity, the correlations were obtained by directly computing correlation coefficients of the 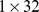 vectors (shown in Table 2). As can be seen, spatial patterns (i.e. projections of the components to the scalp) between the resting and the motor imagery states were very comparable (mean correlation coefficients of 0.95±0.05 and 0.94±0.06 for left and right ICs) for all subjects. The spatial filter, the unmixing vector, was more variable. For example, spatial patterns are highly correlated for Subject 5 with correlation coefficients of 0.92 and 0.96 for the left and right motor IC respectively, however the correlation of spatial filters is very weak (0.09 and 0.34 for left and right ICs). Although the spatial filters might be different, their effectiveness for extracting the motor-related EEG components should be as effective, judging from the similarity of the corresponding spatial patterns. Therefore, in practice, the selection of motor-related components was based on the spatial patterns instead of spatial filters.

**Table 2:** Correlation coefficients of spatial patterns and spatial filters between the resting state and the motor imagery state.

	Left IC		Right IC	
Subjects	Spatial Pattern	Spatial Filter	Spatial Pattern	Spatial Filter
S1	0.99	0.89	0.97	0.71
S2	0.84	0.06	0.87	0.76
S3	0.99	0.80	0.99	0.96
S4	0.93	0.81	0.92	0.54
S5	0.92	0.09	0.96	0.34
S6	0.99	0.90	0.99	0.97
S7	0.95	0.70	0.99	0.93
S8	0.94	0.61	0.91	0.91
S9	0.97	0.93	0.82	0.86
Mean	0.95±0.05	-	0.94±0.06	-

### 2 EEG features induced by motor imagery

**Figure 6:**
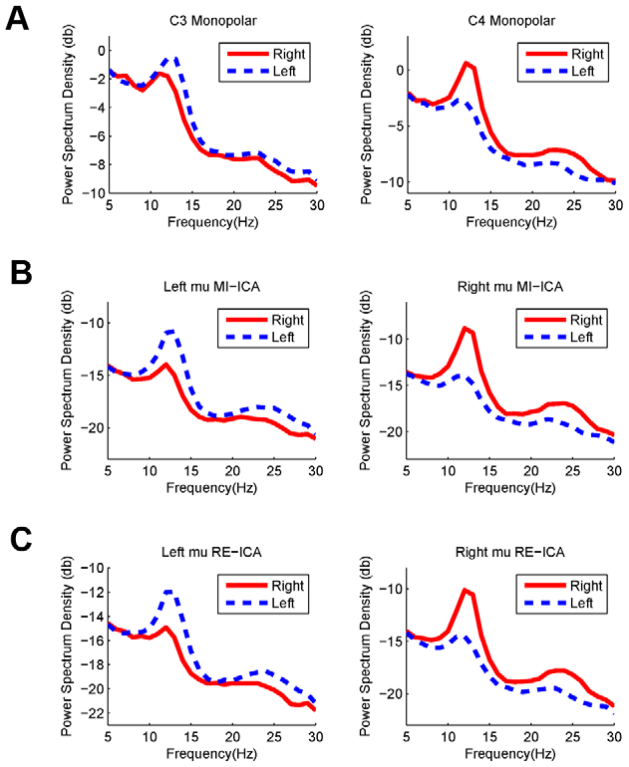
Averaged power spectrum density of EEG signals in motor imagery practice across all subjects. (A) monopolar scalp data at C3 and C4 electrodes; (B) motor-related independent components extracted by ICA using the motor imagery data; (C) motor-related independent components extracted by ICA using the resting data.

Figure 6 shows the PSDs of EEG at C3 and C4 electrodes and the independent motor components after ICA-based filtering using the resting data and the motor imagery data separately. Left- and right-hand motor imagery induced a significant ERD/ERS of the mu/beta rhythms at both channels and of component activations. At C3 and C4 electrodes, spectra of EEG data could be mainly attributed to the background alpha rhythm and the motor related mu/beta rhythms. Because the background alpha activity was not modulated by motor imagery, it might obscure the spectral changes of the mu rhythm. Since ICA can separate neural activities arising from distinct brain processes, it can separate motor-related mu rhythm from the background alpha activity, which in turn could enhance the SNR of motor-imagery induced brain rhythm. As shown in Figure 6, compared to the monopolar scalp data, ICA-based spatial filtering methods significantly enhanced the mean power difference in alpha/beta (8–30 Hz) between the left- and right-hand conditions (Monopolar data: C3/0.63 db and C4/1.24 db, ICA trained with motor imagery data: left motor IC/1.32 db and right motor IC/1.85 db, ICA trained with resting EEG: left motor IC/1.16 db and right motor IC/1.63 db).

### 3 Classification of left- and right-hand imagery movements

The FDA classifier used three different EEG features, PSD of EEG at C3/C4, PSD of independent motor components, and PSD of CSP-filtered EEG, as inputs to classify single-trial motor-imagery movements. Table 3 summarizes the results of 10×10-fold cross-validation. A paired t-test across subjects was used to test the statistical significance of the differences between different feature extraction methods. As expected, compared to the monopolar method, all spatial-filtering methods achieved significantly higher classification accuracies (87.0%, 85.9%, and 86.4% vs. 80.4%, p<0.01). The results of ICA trained with the motor imagery data were slightly better than those trained with the resting data (87.0% vs. 85.9%), but the difference was not statistically significant (p>0.1). The results of using CSP-filtered (based on motor-imagery data) were comparable with those using ICA trained with motor imagery data (86.4% vs. 87.0%, p>0.1) and resting data (86.4% vs. 85.9%, p>0.1). These findings demonstrated the effectiveness of translating ICA-based resting spatial filters to classifying motor imagery EEG data.

**Table 3:** Classification accuracy (%) for all subjects using different feature extraction methods.

	Method			
Subjects	Monopolar	ICA-mi	ICA-rest	CSP
S1	86	84	84	88
S2	66	70	70	72
S3	84	92	92	90
S4	86	94	88	93
S5	84	90	88	88
S6	93	96	96	92
S7	87	92	93	92
S8	85	97	95	95
S9	53	67	68	69
Mean	80.4±12	87.0±9	85.9±11	86.4±9

### 4 Across-session classification of left- and right-hand imagery movements

The session-to-session translation of ICA-based spatial filters was applied to three subjects who participated in two separate BCI experiments on different days. Table 4 shows the classification results for different methods, including the state-to-state and session-to-session methods. Results of ICA-filtered EEG features trained on motor imagery data are the gold standard in the Table. The degradation in classification accuracy was expected for the session-to-session method because of the electrode misalignment and/or long-term non-stationary nature of the EEG. Two major results can be found from the Table. First, the session-to-session translation achieved higher classification accuracy than the monopolar method (91.0% vs. 87.3%). Although the two sessions were recorded on different days with a long interval, spatial patterns of the motor components seemed to be relatively stable, therefore leading to an effective session-to-session translation for improving SNR of the motor activities. Second, the rest-to-work translation outperformed the session-to-session translation on all three subjects (on average, 93.0% vs. 91.0%). For the motor components, the rest-to-work variability in a short-term period should be less of a problem than the long-term session-to-session variability, suggesting that the rest-to-work translation is a viable solution for zero-training of ICA-based spatial filters in a motor imagery-based BCI.

**Table 4:** Classification accuracy (%) of the session-to-session transfer method on three subjects.

	Method			
Subjects	Monopolar	ICA-mi	ICA-rest	Session-to-session
S5	84	90	88	85
S6	93	96	96	95
S8	85	97	95	93
Mean	87.3±5	94.3±4	93.0±4	91.0±5

## Discussion

### 1 ERD/ERS and independent motor components

**Figure 7:**
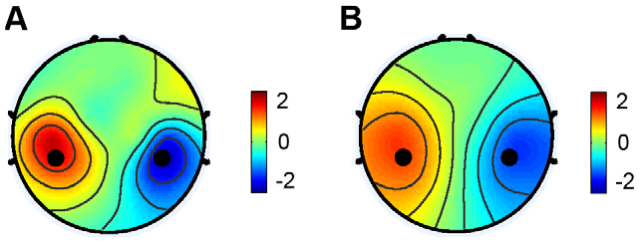
Spatial distributions of EEG power difference and IC spatial pattern difference. (A) power difference between left- and right-hand motor imagery conditions; (B) difference of spatial patterns between left and right independent motor components obtained from the motor imagery data.

To make a rest-to-work filter translation effective, scalp distribution of ERD/ERS during motor imagery needs to be consistent with spatial patterns of the independent motor components. Suppose that motor imagery of the hand movement induces similar ERD on the contralateral hemisphere, as well as ERS on the ipsilateral hemisphere, the scalp distribution of the power difference between left and right hand movements should be similar to the difference of spatial patterns between the left and right motor components. To verify this hypothesis, we calculated the spatial distribution of power difference (8–30 Hz) between the left and the right hand motor imagery across all subjects. Figure 7A shows the scalp distribution of the power changes. In addition, the difference between spatial patterns of the left and right motor components was also computed for comparison (Figure 7B). Both spatial distributions are widespread over the sensorimotor areas with C3 and C4 electrodes located near the center of two lateral sub-regions. Correlation between these two distributions was very high (r = 0.90). The difference of ICA spatial patterns has a more widespread distribution over the scalp, indicating that the motor components originate from multiple subareas of the sensorimotor cortex, whereas hand motor imagery might only modulate subcomponents of the motor rhythms corresponding to the hand areas. Due to the fact that the hand areas are the largest parts in the sensorimotor areas, spatial filters optimal for extracting independent motor components can be used as estimates of spatial filters during motor imagery of hand movements.

### 2 Comparison of ICA and CSP

In the proposed state-to-state translation method, this study used ICA to find spatial filters mainly because of its advantage in unsupervised learning. In previous studies, the CSP method has been more commonly used for classifying motor imagery EEG due to its simplicity in computation and high performance in classification [33]. The classification results of ICA and CSP in this study showed very close performance when using the filtered EEG power between 8 and 30 Hz (87.0% and 86.4%). Robustness of ICA depends on the size of data and ICA has much larger computational cost, therefore, CSP is more feasible for online application when labeled training data are available. However, because no labeled data were available in the resting EEG data, CSP is not practical for the proposed rest-to-work translation method.

In the session-to-session approach, both ICA and CSP methods are applicable. Several recent studies have employed the CSP method to derive zero-training spatial filters [9]. This study demonstrated that ICA could also be used for translating spatial filters from session to session. On three subjects, the session-to-session translation of spatial filters achieved a significant improvement in classification accuracy, compared to the method using monopolar EEG data (91.0% vs. 87.3%). For all three subjects, the state-to-state method outperformed the session-to-session method (93.0% vs. 91.0%). It is worth pointing out that the performance of the session-to-session method heavily depended on the alignment of electrode locations from one session to another. The placement of the electrodes was carefully aligned across sessions in this study. The performance of session-to-session filter translations could have been much worse if the placement was not perfectly aligned. On the other hand, the rest-to-work filter translation is much less sensitive to the misalignment of the electrodes.

### 3 Online implementation

**Figure 8:**
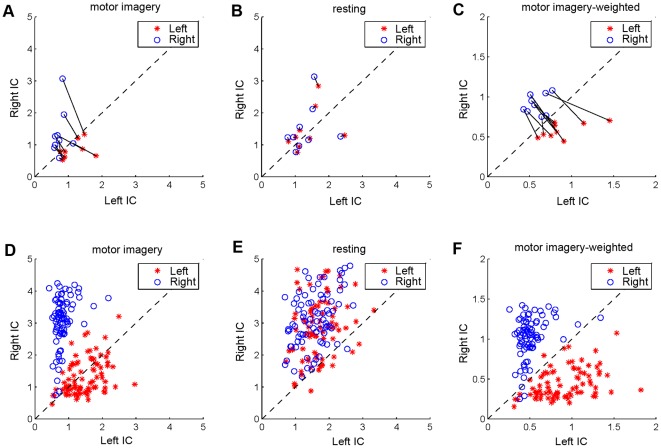
EEG power of motor ICs during resting and motor imagery states. (A) EEG power of motor ICs during motor imagery; (B) EEG power of motor ICs during resting; (C) Weighted EEG power of motor ICs during motor imagery (original power divided by the mean power of the resting data). (D) Single-trial EEG power of motor ICs during motor imagery on one subject. (E) Single-trial EEG baseline power of motor ICs during resting. (F) Weighted single-trial EEG power of motor ICs during motor imagery. In (A), (B), and (C), each solid line connects left hand and right hand data for a subject. The dash line indicates the line 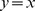.

In this study, the proposed rest-to-work translation method was demonstrated by offline analysis using data recorded during online BCI sessions. Toward an online implementation of the proposed method, three specific issues need to be addressed:

1. Data recording: In this study, the resting data comprised interleaved data segments corresponding to the resting periods across multiple trials. For the purpose of an online rest-to-work translation, the resting data need to be recorded before a BCI session. For example, few minutes of data recorded during a resting state can be used for running ICA to obtain spatial filters. Because no mental tasks need to be involved during resting data recording, this procedure will not increase much of complexity of the system use. The spatial filters derived from the resting data can be used in the subsequent online BCI sessions for improving the system performance.
2. Computational cost: To make the proposed method practical, the ICA-based processing needs to be completed with a reasonable amount of time before a BCI session. The interval between recording resting data and the subsequent online BCI session must be long enough for ICA to converge to spatial filters. In this study, the ICA processing was performed using Matlab (Mathworks Inc.) on a workstation with Intel Xeon w3520@2.67GHz quad processors. This procedure took about 60 seconds, making the total time for obtaining the ICA-based filters to be few minutes. Using a high-performance computer or other technologies such as parallel or cluster computing could further reduce the computation time.
3. Zero-training classifier: The implementation of the FDA classifier in an online motor-imagery BCI requires some labeled training samples to optimize the parameters of the classifier. Figure 8A plots the EEG power of left and right motor components under left- and right-hand imagery movements, which shows a significant asymmetry over two hemispheres (Left IC and Right IC). Under this circumstance, it is difficult to optimize a zero-training classifier when labeled data are not available. However, the hemispheric asymmetry might be relatively stable in the resting state compared to the motor imagery state. Therefore, using EEG power of the resting data (Figure 8B) as the baseline to calculate a weighted power of each component (i.e., divided by the mean power of the resting data) could result in a refined classifier. As shown in Figure 8C, after the weighting process, the classification could be performed by simply comparing the EEG power between the left and the right motor IC. The zero-training classifier can be described as follows:
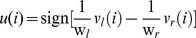
(8)where 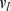 and 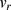 are EEG power of the left and right motor ICs in a single trial (*i*) of motor imagery, 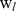 and 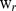 are precalculated mean power of the two ICs during the resting state. The classifier (*u*) returns +1 or −1 corresponding to motor imagery of the left or the right hand movement respectively. In practice, the classifier could be combined with the state-to-state spatial filters to implement a zero-training BCI system. Figure 8D, E, F shows an example of this process on a subject. For this subject, EEG power of the right motor IC is significantly higher than that of the left motor IC; therefore, the weighting process significantly improved classification performance of the zero-training classifier from 69.4% to 96.9%. Across all subjects, the zero-training classifier achieved a significant performance improvement from 69.2±14% to 83.1±12%, which is very close to the classification accuracy when using FDA (85.9±11%, cf. Table 3).

### 4 Classification with multiple motor ICs

**Figure 9:**
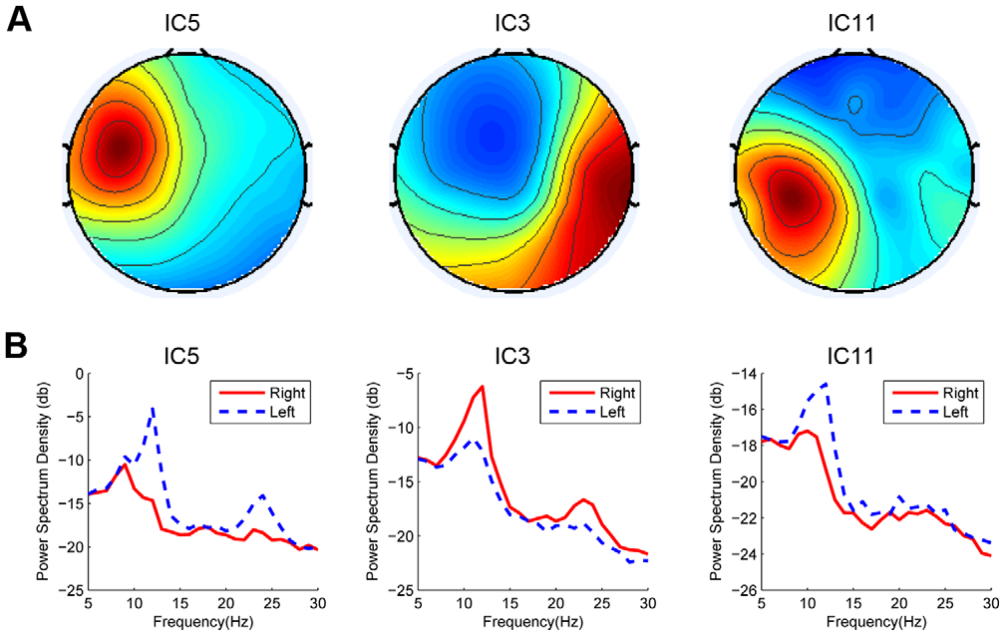
Spatial patterns and averaged PSDs of the three motor ICs for Subject 5. (A) Spatial patterns; (B) Averaged PSDs in motor imagery practice.

ICA extracted three motor ICs (two on the left hemisphere) for Subject 5. Figure 9A shows the scalp maps of the three motor ICs for the resting condition. After applying the corresponding spatial filters to the motor imagery data, the PSDs for three ICs under Left and Right motor imagery conditions could be obtained (Figure 9B). The *r* square values (i.e., the correlation between EEG features and task labels [4]) for the band-pass power (8–30 Hz) of the three ICs are: 0.25, 0.42, and 0.20, indicating a significant difference between the left and right imagery conditions for all the motor ICs.

In general, the involvement of all motor ICs might improve classification performance through using feature combination approaches. However, classification accuracy using with three motor ICs was comparable with that using two motor ICs (86.6% vs. 87.8%). The reason that the involvement of all ICs did not achieve performance improvement lies in the following aspects: (1) the features from the two left ICs are highly correlated with each other (r = 0.70); (2) the generalization ability of the classifier decreases when the feature dimension in classification increases. Because this study had only one subject with more than two motor ICs, the approach for combining multiple motor ICs to improve classification performance requires further investigation.

### 5 Further improvement

According to the finding that spatial patterns of the motor ICs under different mental conditions within a short-term period are relatively stable, this study simply adapted the two ICA-based spatial filters derived from the resting state to the motor imagery state for enhancing EEG changes induced by the motor imagery. Although effectiveness of the resting-to-work translation of spatial filters has been demonstrated in this study, there are several ways to improve the method. First, this study used ICA to decompose the motor components from 32-channel EEG data. The SNR of the mu/beta rhythms could be further improved by using high-density EEG recordings with more electrodes. Second, other EEG spatial filters such as beamformers [34] could be trained by using the topographies of the motor ICs obtained from the resting data as spatial constrains. System performance could be further improved through applying feature combination techniques and ensemble classification methods to integrate information from different types of spatial filters.

### 6 Other applications

This study implemented a rest-to-work translation of spatial filters for a motor imagery-based BCI. The basic principle of the proposed method can be further extended to a state-to-state translation in other applications. First, it improves data processing in data poor environments. Because the size of task-related data is always limited in BCI studies, a state-to-state translation can make it possible to combine task-irrelevant (e.g., resting) data for applying advanced data processing techniques such as the ICA-based spatial filtering technique. Second, a more general translation between different sensory systems (e.g., visual and sensorimotor systems) might be possible. For example, in a hybrid BCI system [35] where motor imagery and visual attention are employed at the same time, translating spatial filters of both the motor imagery data and the visual attention data from one state to another might be helpful for facilitating user training. In addition, the state-to-state translation might be helpful for contrasting information between different tasks, which involve common resources in the same sensory system. For example, data of motor imagery of hand movements can be used to construct a new classifier to discriminate not only the two states of imagining hand movements, but also the idling state or the motor imagery state of foot movement [36].

### 7 Conclusion

This study proposed a rest-to-work translation of ICA-based spatial filters for classifying single-trial EEG during motor imagery of hand movements. Spatial filters derived from the resting data and the motor imagery data showed very similar spatial patterns and spectral profiles, verifying the hypothesis that spatial brain patterns of the sensorimotor system are relatively stable under the two different states. The spatial filters derived from the resting EEG data were proved effective for improving the SNR of the motor imagery induced EEG changes. Furthermore, spatial filters derived from ICA based on resting and BCI practice states provided comparable classification accuracies in discriminating left- and right-hand motor imagery (87.0% and 85.9%). Finally, a comparison study between the state-to-state translation and the session-to-session translation demonstrated the superiority of the proposed rest-to-work translation method (93.0% vs. 91.0%). In summary, this study proposed and demonstrated a new state-to-state translation method for optimizing EEG spatial filters using readily available and non-labeled resting data, which could considerably increase the practicality of online BCI systems.
